# d-Idose, d-Iduronic Acid, and d-Idonic Acid from d-Glucose via Seven-Carbon Sugars

**DOI:** 10.3390/molecules24203758

**Published:** 2019-10-18

**Authors:** Zilei Liu, Sarah F. Jenkinson, Akihide Yoshihara, Mark R. Wormald, Ken Izumori, George W. J. Fleet

**Affiliations:** 1 Chemistry Research Laboratory, Department of Chemistry, University of Oxford, Oxford OX1 3TA, UK; zileiliu@scripps.edu (Z.L.); sarah.barker@sjc-oxford.com (S.F.J.); 2 Oxford Glycobiology Institute, Department of Biochemistry, University of Oxford, Oxford OX1 3QU, UK; mark.wormald@bioch.ox.ac.uk; 3 International Institute of Rare Sugar Research and Education, Kagawa University, Kagawa 761-0795, Japan; yoshihara@ag.kagawa-u.ac.jp (A.Y.); izumori@izumoring.com (K.I.)

**Keywords:** rare sugar, D-idose, D-iduronic acid, D-idonic acid, monosaccharide

## Abstract

A practical synthesis of the very rare sugar d-idose and the stable building blocks for d-idose, d-iduronic, and d-idonic acids from *ido*-heptonic acid requires only isopropylidene protection, Shing silica gel-supported periodate cleavage of the C6-C7 bond of the heptonic acid, and selective reduction of C1 and/or C6. d-Idose is the most unstable of all the aldohexoses and a stable precursor which be stored and then converted under very mild conditions into d-idose is easily prepared.

## 1. Introduction

**Scheme 1:**
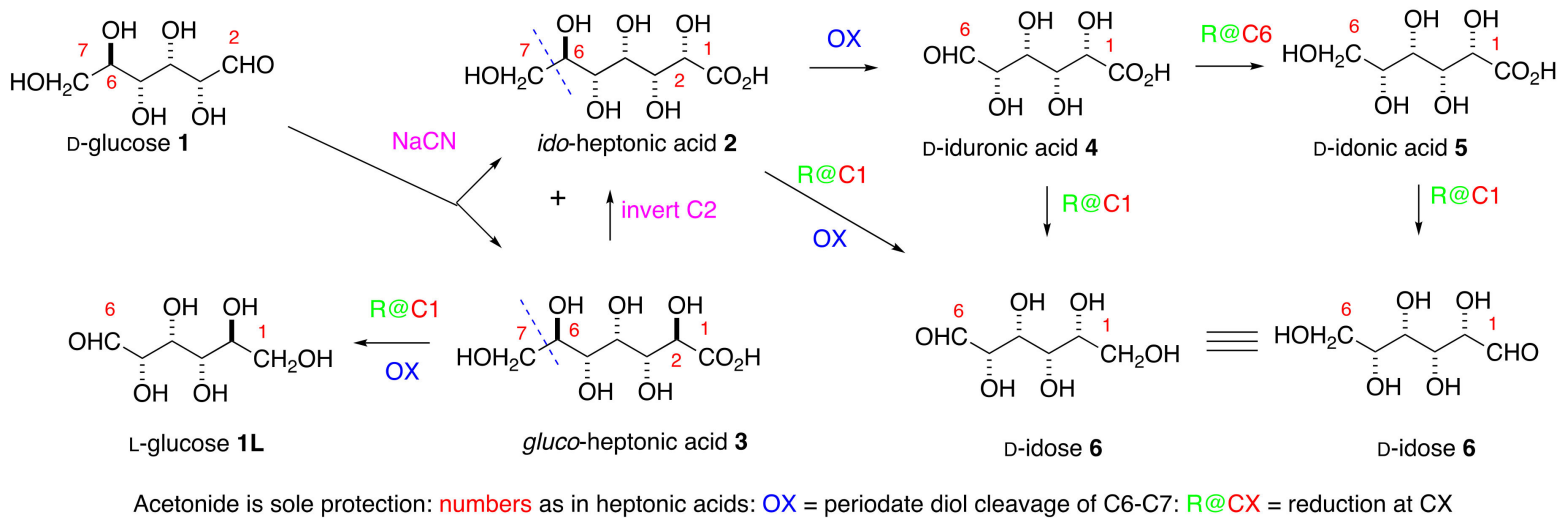
Strategy for the synthesis of d-iduronic 4 and d-idonic 5 acids, and d-idose 6.

Although the biotechnology of Izumoring [1,2] and related approaches [3] have revolutionized the availability of large quantities of many rare sugars, the practical conversion of d-sorbose to d-idose **6** by xylose isomerase [4] is beset by the 97:3 equilibrium in favor of d-sorbose and cannot easily produce significant quantities of d-idose **6** [5]. d-Idose, the rarest of all rare hexoses [6,7,8], is the least stable to acid, base, or heat; idose is the only aldohexose to never be crystallized. The easy preparation of a stable precursor that could be converted to d-idose under very mild conditions, would simplify both the investigation of its biological properties and its incorporation into oligosaccharides and aglycone pharmacophores. The Kiliani reaction [9] of d-glucose **1** with sodium cyanide, to give the sodium salts of the epimeric d-*ido* (**2**) and l-*gluco*- (**3**) heptonic acids in a ratio of approximately 1:4, has been a massive industrial process for many decades (Scheme 1) [10]. 

Salts of the *gluco*-acid **3**, available cheaply [11] in >99% purity, are used as chelating agents, detergents, and for many other purposes. The mother liquors after the first crystallization contain more of the *ido*-acid **2** mixtures, such as Seqlene-50 [12] consisting of a 1:1 ratio of **2** and **3** at 50% concentration, are very cheap industrial cleaners.

Acetonation is the only protection needed for the synthesis of d-idose **6**, d-iduronic **4**, and d-idonic **5** acids. The key steps include (i) the Shing [13] periodate cleavage of the C6-C7 bond in heptonic acids **2** and **3**, and the (ii) adjustment of the oxidation levels at C1 and C6 in the heptonic acid [Scheme 1]. The C_2_ symmetry of the *ido*-motif means that the aldehyde in d-idose may derived from either C1 or C6 of the *ido*-heptonic acid **2**. Efficient inversion at C2 of *gluco*-acid **3** allows the unambiguous synthesis of the d-*ido* targets in the paper without any separation of diastereomers. Preliminary studies of the use of Seqlene to access acetonides of **2** are reported.

**Scheme 2:**
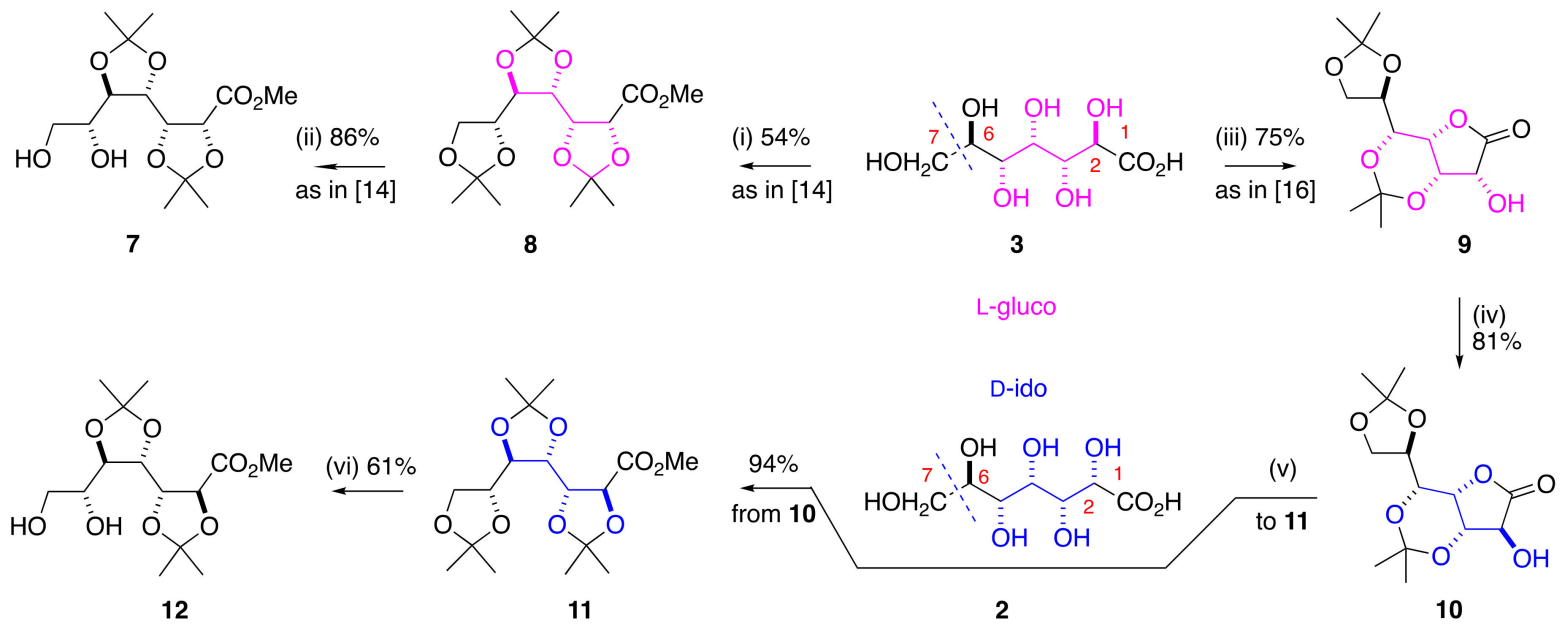
(**i**) Me_2_C(OMe)_2_, HCl, MeOH, reflux, 2 h (54%) [14]; (**ii**) MeOH, H_2_SO_4_, (86%) [14]; (**iii**) Me_2_CO, H_2_SO_4_ (75%) [16]; (**iv**) (CF_3_SO_2_)_2_O, pyridine, THF; then CF_3_CO_2_Na, DMF (81%) [19]; (**v**) Me_2_C(OMe)_2_, HCl, MeOH, reflux, 2 h (94%); (**vi**) MeCO_2_H:H_2_O:MeOH, 2:1:3, 40 °C, 5.5 h (61%).

A similar strategy has been used for the scalable synthesis of l-glucose **1L** from *gluco*-acid **3** with no column chromatography [14]. Treatment of the sodium salt of **3** with 2,2-dimethoxypropane in methanolic HCl formed the triacetonide **8** (54%) (Scheme 2); selective hydrolysis of **8** gave diol **7** (86%) and, in four steps, l-glucose **1L** in 80% yield. The triacetonide **8** was also efficiently converted to many other rare sugars with differing oxidation levels at C1 and C6 [15]. 

## 2. Results and Discussion

### 2.1. Acetonides of Heptonic Acids and Inversion of Configuration at C2 in gluco-Acid ***3***

Access to the d-*ido*-diol **12**, the intermediate for all the d-*ido* sugars, required inversion of configuration at C2 of the *gluco*-heptonic acid **3** (Scheme 2). The *gluco*-diacetonide **9** [16] formed a stable triflate (88%) [17], which, on treatment with trifluoroacetate [18], gave the diacetonide of *ido*-heptonolactone **10** (81%) [19].

Treatment of the *ido*-diacetonide **10** with 2,2-dimethoxypropane in methanolic hydrogen chloride formed the *trans,trans*-triacetonide **11** (94%) with all the substituents on the dioxolane ring *trans* to each other. Although there are other possible diacetonides of **11** that contain different size rings, the ^13^C singlet carbons at δ 109.5, 109.6, and 111.5 clearly showed all the isopropylidene protecting groups were 5-ring ketals [20].

Seqlene-50 contains a 50% concentration of an aqueous solution of a 1:1 mixture of the sodium salts of the epimeric heptonic acids **2** and **3**. Evaporation of Seqlene to dryness, followed by treatment of the dark brown residue with 2,2-dimethoxypropane in methanolic hydrogen chloride, gave a 1:1 inseparable mixture of the triacetonides of the methyl *ido*- (**11**) and *gluco*- (**8**) heptonates (36%); the ratio was estimated by comparison of ^1^H NMR of the mixture to those of pure samples of the triacetonides **11** and **8**]. It has been reported [21] that the double cadmium salt of the *ido*-heptonic acid **2**, cadmium d-*glycero*-d-*ido*-heptonate cadmium chloride monohydrate, can be crystallized from the Seqlene 1:1 mixture; in a preliminary study of one crystallization as cadmium salts, the ratio of the **11**:**8** esters improved to 3:1. If large amounts of **11** are needed, it may be that optimization of this crystallization will be of considerable benefit.

Hydrolysis of the terminal acetonide in the presence of other acetonides usually proceeds in high yield; for example, partial hydrolysis of the *gluco*-triacetonide **8** by sulfuric acid in methanol gave 86% of the diol **7** on a large scale. More care is needed in the removal of the terminal acetonide in **11** where treatment with acetic acid:water:methanol, 2:1:3, afforded the key *ido*-heptonate **12** in 61% yield.

### 2.2. d-Idose, d-iduronic, and d-idonic Acids from Protected Diol Ido-Heptonate ***12***

**Scheme 3:**
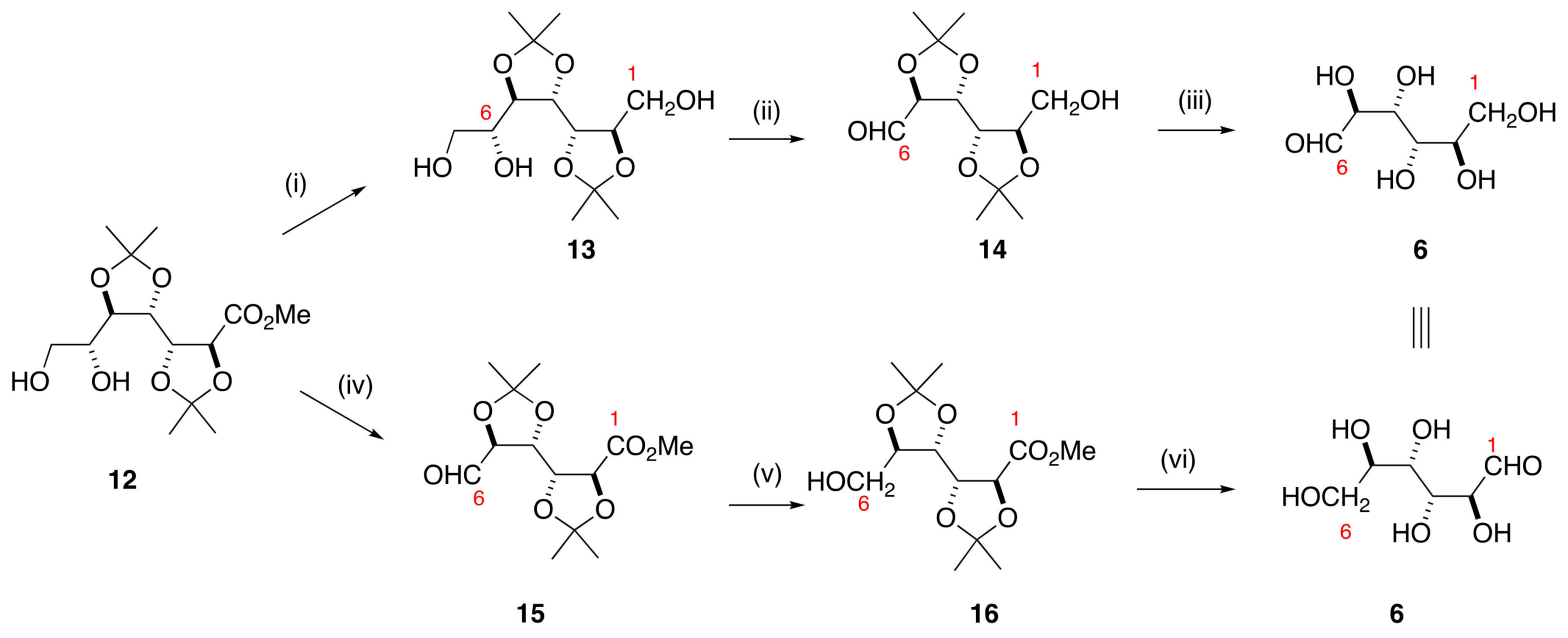
(**i**) NaBH_4_, MeOH, 0 °C, 3 h (95%); (**ii**) NaIO_4_, CH_2_Cl_2_, rt, 2 h (100%); (**iii**) DOWEX^®^ 50WX8-200, 24 h (97%) (**iv**) NaIO_4_, CH_2_Cl_2_, rt, 1 h (89%); (**v**) NaBH_4_, MeOH, 0 °C, 1 h (63%); (**vi**) DIBALH, CH_2_Cl_2_, −78 °C, 2 h; then DOWEX^®^ 50WX8-200, 24 h (76%).

Reduction of the diol ester **12** by sodium borohydride in methanol gave the triol **13** (95%) (Scheme 3). Oxidative cleavage of C6-C7 bond in **13** by silica gel-supported sodium periodate in dichloromethane [13] formed the aldehyde **14** in quantitative yield. Subsequent hydrolysis of the acetonide protecting groups with DOWEX^®^ resin gave d-idose **6** (97% from **13**; 53% from **11**) with identical ^13^C and ^1^H NMR spectra to those of an authentic sample, the purity of which was established by HPLC; the spectra are consistent with those previously reported for d-idose [22,23]. In this sequence, C1 and C6 of d-idose **6** were derived respectively from C6 and C1 of the *ido*-heptonic acid **2**. The triol **13**, a white crystallizable solid, is an ideal stable precursor for generation of d-idose. 

Initial oxidation of **12** with silica gel-supported sodium periodate in dichloromethane gave the protected d-iduronic acid **15** (89%). Sodium borohydride in methanol at 0 °C caused selective reduction of the aldehyde group to give **16** (63%) as a protected d-idonic acid **2**. Further reduction of the ester **16** with diisobutylaluminum hydride (DIBALH) in toluene gave the aldehyde **14** from which the acetonide groups were removed by DOWEX resin to produce d-idose **6** (76% from **16**), identical to the sample formed above; in contrast to the first route, C1 and C6 of d-idose **6** were derived respectively from C1 and C6 of the *ido*-heptonic acid **2**.

## 3. Experimental

### 3.1. General Experimental

All commercial reagents were used as supplied. Solvents were used as supplied (analytical or HPLC grade), without prior purification. Thin-layer chromatography (TLC) was performed on aluminium sheets coated with 60 F_254_ silica. Plates were visualized using a 0.2% *w*/*v* cerium (IV) sulfate and 5% ammonium molybdate solution in 2 M sulfuric acid. Melting points were recorded on a Kofler hot block and are uncorrected. Optical rotations of the protected sugars were recorded on a Perkin-Elmer 241 polarimeter with a path length of 1 dm; concentrations are quoted in g 100 mL^−1^. Optical rotations were recorded on a Jasco R1030 polarimeter, Na^+^ lamp, (Jasco, Tokyo, Japan) at 20 °C, polarimeter with a path length of 1 dm. Infrared spectra were recorded on a Perkin-Elmer 1750 IR Fourier transform spectrophotometer using thin films on a diamond ATR surface (thin film). Only the characteristic peaks are quoted. Nuclear magnetic resonance (NMR) spectra were recorded on a Bruker AMX 500 (^1^H: 500 MHz and ^13^C: 125.7 MHz) or Bruker AVIII 400 HD nanobay and Bruker DQX 400 (^1^H: 400 MHz, ^13^C: 100.6 MHz and ^19^F: 375 MHz) or Bruker DPX 250 (^1^H: 250 MHz and ^13^C: 62.5 MHz) or Varian Mercury 300 (^1^H: 300 MHz, ^13^C: 75 MHz) spectrometers in the deuterated solvent stated. ^1^H and ^13^C NMR spectra were assigned by utilizing 2D COSY, HSQC and HMBC spectra. All chemical shifts (δ) are quoted in ppm and coupling constants (*J*) in Hz. Residual signals from the solvents were used as an internal reference. For solutions in D_2_O acetonitrile was used as an internal reference. HRMS measurements were made using a micro-time-of-flight (TOF) mass analyzer using electrospray ionization (ESI) or an HP 5988A mass spectrometer using chemical ionization (CI). The purity of d-idose by high-performance liquid chromatography (Hitachi GL-611 column, Tokyo, Japan, and Shimadzu RID-6A refractive index detector, Kyoto, Japan) at 60 °C, eluted with 10^−4^ M NaOH at a flow rate of 1.0 mL/min. Seqlene-50 was obtained from Hallstar Engineering of Chicago, IL,USA (see Supplementary Materials for ^1^H and ^13^C NMR spectra).

### 3.2. Methyl 2,3:4,5:6,7-Tri-O-Isopropylidene-d-glycero-d-ido-Heptonate ***11***

Method 1 from Ido-Heptono-1,4-Lactone **10**

A methanolic solution of hydrogen chloride [prepared by dropwise addition of acetyl chloride (0.8 mL, 11.2 mmol) to methanol (6.6 mL) under argon at 0 °C] was added to a solution of the *ido*-diacetonide **10** (3.45 g, 12.0 mmol) in 2,2-dimethoxypropane (50 mL). The reaction mixture was then refluxed for 2 h when TLC (cyclohexane/ethyl acetate, 1:1) showed the formation of a major product (*R*_f_ 0.70). Sodium carbonate (5 g) was added into reaction mixture to neutralize the pH to 7 (the color of reaction mixture turned from brown to light yellow). After the solids were removed by filtration, the solvent was removed in vacuo to give a residue that was dissolved into cyclohexane (50 mL). The solution was washed with distilled water (3 × 50 mL), dried (MgSO_4_) and solvent was removed in vacuo to yield pure triacetonide **11** (3.10 g, 8.6 mmol, yield 71%). Further extraction of the aqueous layer by ethyl acetate (3 × 50 mL) gave partially acetonated products; removal of solvent in vacuo, afforded a residue (~2.0 g), which was dissolved into acetone (20 mL), and the procedures above were repeated to obtain more **11** (4.10 g in total, 95%) as a syrup, which used in the next step without further purification.

Method 2 from Seqlene without Recrystallization 

A solution of Seqlene (2 mL) was fully dried in vacuo to give a dark brown residue (~1.3 g). A methanolic solution of hydrogen chloride (prepared by dropwise addition of acetyl chloride (0.4 mL, 5.6 mmol) to methanol (3.3 mL) under argon at 0 °C) was added to a solution of the residue in 2,2-dimethoxypropane (25 mL). The reaction mixture was then refluxed for 5 h when TLC (cyclohexane/ethyl acetate, 1:1) showed the formation of a major product (*R*_f_ 0.70). Sodium carbonate (2 g) was added to the reaction mixture to neutralize the pH to 7 (the color of reaction mixture turned from brown to light yellow). After the solids were removed by filtration, the solvent was removed in vacuo to give a residue that was dissolved into cyclohexane (20 mL). The solution was washed with distilled water (3 × 10 mL), dried (MgSO_4_) and solvent was removed in vacuo to yield a mixture of **8** and **11** (680 mg in total, 36%) as a NMR ratio of 1:1 (some impurities were also detected).

Method 3 (from Seqlene with Recrystallization)

A solution of Seqlene (10 mL, ~7.2 g solid) was diluted with water (60 mL) and passed through a column containing Amberlite IR-120 (H^+^) cation-exchange resin column (~40 mL). The eluent was stirred at 100 °C for 5 h with cadmium carbonate (2.7 g). Then, cadmium chloride (2.9 g) was added. After filtration by active carbon, the solution was concentrated in vacuo to half of the volume. Ethanol (~40 mL) was added until the solution became muddy. The solution was left to recrystallize at rt overnight to obtain a cadmium salt (1.5 g) of lactones that was subjected to the protection conditions as shown above to form a mixture of triacetonide **8** and **11** (2.0 g) as a ratio of 3:10 according to ^1^H NMR.

HRMS *m*/*z* (ESI + ve): found 383.1673 [M + Na]^+^, C_17_H_28_O_8_Na^+^ requires 383.1676; [α]_D_^20^ + 18.9 (*c* 1.25, MeOH); ν_max_ (thin film): 1764 (s, C=O); δ_H_ (CD_3_OD, 400MHz): 1.22 (3H, s, CH_3_), 1.28 (3H, s, CH_3_), 1.29 (6H, s, 2 x CH_3_), 1.35 (3H, s, CH_3_), 1.36 (3H, s, CH_3_), 3.69 (3H, s, OCH_3_), 3.82–3.85 (1H, m, H7), 3.92–3.94 (1H, m, H5), 4.00 (1H, dd, H4, *J*_4,3_ 2.1, *J*_4,5_ 7.5), 4.01–4.04 (2H, m, H6, H7′), 4.17 (1H, dd, H3, *J*_3,4_ = 2.1, *J*_3,2_ 7.8), 4.53 (1H, d, H2, *J*_2,3_ 7.8), δ_C_ (CD_3_OD, 100MHz) 24.1 (CH_3_), 24.8 (CH_3_), 25.5 (CH_3_), 25.7 (CH_3_), 26.2 (CH_3_), 26.6 (CH_3_), 51.5 (OCH_3_), 67.1 (C7), 75.3 (C2), 77.0 (C5), 77.2 (C6), 77.9 (C4), 78.6 (C3), 109.5 (C(CH_3_)_2_), 109.6 (C(CH_3_)_2_), 111.5 (C(CH_3_)_2_), 171.3 (C1); *m*/*z* (ESI + ve): 383 ([M + Na]^+^, 100%).

### 3.3. Methyl 2,3:4,5-Di-O-Isopropylidene-d-glycero-d-ido-Heptonate ***12***

A solution of triacetonide **11** (4.10 g, 11.4 mmol) in acetic acid:water:methanol (15 mL, 2:1:3) was stirred at 40 °C for 5.5 h until TLC (ethyl acetate) showed the formation of one major product (*R*_f_ 0.60). The reaction mixture was concentrate in vacuo to ~2 mL and then stirred with NaHCO_3_ (sat. aq, 40 mL). Cyclohexane (3 × 40 mL) was used to extract the unreacted starting material **11** (~2.2 g) on which the process was repeated. The aqueous layer was then washed with dichloromethane (3 × 40 mL), the combined extracts dried (MgSO_4_), and the solvent removed in vacuo to obtain **12** (1.20 g, 32%) as a clear oil. The unreacted **11** from cyclohexane was recycled by the hydrolysis protocol to obtain more **12** (2.21 g, 61% based on recovered **12**).

HRMS *m*/*z* (ESI + ve): found 343.1360 [M + Na]^+^, C_14_H_24_O_8_Na^+^ requires 343.1363; [α]_D_^20^ + 63 (*c* 0.46, MeOH); ν_max_ (thin film): 3469 (br, OH), 1760 (s, C=O); δ_H_ (CDCl_3_, 400MHz) 1.44 (3H, s, CH_3_), 1.45 (3H, s, CH_3_), 1.47 (3H, s, CH_3_), 1.51 (3H, s, CH_3_), 2.42 (1H, t, OH7, *J*_OH,H7_ = *J*_OH,H7′_ 5.5), 2.98 (1H, d, OH6, *J*_OH, H6_ 5.3), 3.72–3.79 (2H, m, H6, H7), 3.83 (3H, s, OCH_3_), 3.84–3.87 (1H, m, H7′), 4.12 (1H, t, H5, *J*_5,6_ = *J*_5,4_ 7.3), 4.26 (1H, dd, H4, *J*_4,3_ 3.1, *J*_4,5_ 7.3), 4.33 (1H, dd, H3, *J*_3,4_ 3.1, *J*_3,2_ 7.6), 4.70 (1H, d, H2, *J*_2,3_ 7.6); δ_C_ (CDCl_3_, 100MHz) 26.0 (CH_3_), 26.6 (CH_3_), 26.7 (CH_3_), 27.4 (CH_3_), 52.6 (OCH_3_), 63.9 (C7), 74.7 (C4), 73.0 (C6), 75.5 (C2), 76.8 (C5), 78.4, 78.6 (C3 and C4), 110.0 (C(CH_3_)_2_), 111.6 (C(CH_3_)_2_), 171.4 (C1); *m*/*z* (ESI + ve): 343 ([M + Na]^+^, 100%).

### 3.4. 2,3:4,5-Di-O-Isopropylidene-d-glycero-d-ido-Heptitol ***13***

Sodium borohydride (334 mg, 8.80 mmol) was added into a solution of **12** (1.41 g, 4.40 mmol) in methanol (20 mL) at 0 °C, and the solution was stirred for 3 h at rt until TLC (ethyl acetate) showed the consumption of starting material (*R*_f_ 0.57) and the formation of a new product (*R*_f_ 0.37). Acetic acid (~0.5 mL) was added into the solution to adjust the pH to 7, and the solvent was removed in vacuo to obtain a crude product which was further purified by flash column chromatography (ethyl acetate/methanol, 50:1) to obtain the triol **13** as a white solid (1.20 g, 95%).

HRMS *m/z* (ESI + ve): found 315.1415 [M + Na]^+^, C_13_H_24_O_7_Na^+^ requires 315.1414; m.p. 92 °C–94 °C; [α]_D_^20^ + 64 (*c*, 0.68 in MeOH); ν_max_ (thin film): 3389 (broad, OH); δ_H_ (CD_3_OD, 400MHz) 1.38 (3H, s, CH_3_), 1.40 (9H, s, 3 x CH_3_), 3.55 (1H, dd, H7, *J*_7,6_ 6.3, *J*_gem_ 11.0), 3.60–3.53 (1H, m, H6), 3.65 (1H, dd, H1, *J*_1,2_ 5.2, *J*_gem_ 11.9), 3.71 (1H, dd, H1′, *J*_1′,2_ 4.0, *J*_gem_ 11.9), 3.73 (1H, dd, H7′, *J*_7′,6_ 2.9, *J*_gem_ 11.0), 3.99 (1H, dd, H3, *J*_3,4_ 1.5, *J*_3,2_ 8.5), 4.03–4.05 (2H, m, H4, H5), 4.18 (1H, ddd, H2, *J*_2,1′_ 4.1, *J*_2,1_ 5.2, *J*_2,3_ 8.5); δ_C_ (CD_3_OD, 100MHz) 25.8 (2 x CH_3_), 26.2 (CH_3_), 26.3 (CH_3_), 61.7 (C1), 63.6 (C7), 73.8 (C6), 76.6, 78.5 (C4, C5), 77.4 (C3), 77.9 (C2), 108.9 (C(CH_3_)_3_), 109.2 (C(CH_3_)_2_); *m*/*z* (ESI + ve): 315 ([M + Na]^+^, 100%).

### 3.5. Methyl 2,3:4,5-Di-O-Isopropylidene-d-glycero-d-Iduronate 15 and Methyl 2,3:4,5-Di-O-Isopropylidene-d-glycero-d-Idonate ***16***

Silica gel-supported NaIO_4_ (4.50 g) was added portionwise to a vigorously stirred solution of **12** (791 mg, 2.47 mmol) in dichloromethane (30 mL). After 1 h, TLC analysis (cyclohexane/ethyl acetate, 1:1) showed no remaining starting material (*R*_f_ 0.19) and formation of a single product (*R*_f_ 0.70). The mixture was filtered and the silica gel was thoroughly washed with CH_2_Cl_2_ (4 × 30 mL). The solvents were removed in vacuo to afford the crude aldehyde **15** (630 mg, 89%). Then, sodium borohydride (20.9 mg, 1.36 mmol) was added to a solution of the crude aldehyde **15** in methanol (5 mL) at 0 °C. The reaction mixture was stirred at 0 °C for 1 h until TLC (cyclohexane/ethyl acetate, 1:1) indicated the formation of one major product (*R*_f_ 0.53) and a minor product (0.30). After acetic acid (~0.2 mL) was added into the reaction mixture to adjust pH to 7, ethyl acetate (3 × 10 mL) was used to extract the product and organic layer was dried (MgSO_4_), filtered and the solvent was removed to obtain a residue which was further purified by flash column chromatography (cyclohexane/ethyl acetate, 1:3) to obtain the major product **16** as a clear oil (410 mg, 57% 2 steps).

HRMS *m*/*z* (ESI + ve): found 343.1362 [M + Na]^+^, C_14_H_24_O_8_Na^+^ requires 343.1363; [α]_D_^20^ + 33 (*c* 1.02, CHCl_3_); ν_max_ (thin film): 3495 (br, OH), 1759 (s, C=O); δ_H_ (CDCl_3_, 400MHz) 1.46 (3H, s, CH_3_), 1.47 (3H, s, CH_3_), 1.48 (3H, s, CH_3_), 1.50 (3H, s, CH_3_), 2.11 (1H, br-dd, OH6, *J*_OH,H6′_ 5.0, *J*_OH,6_ 7.0), 3.71 (1H, ddd, H6, *J*_6,5_ 4.0, *J*_6,OH_ 7.3, *J*_gem_ 12.0), 3.82 (3H, s, OCH_3_), 3.88 (1H, dt, H6′, *J*_6′,5_ = *J*_6′,OH_ 4.0, *J*_gem_ 12.0), 4.15 (1H, dd, H4, *J*_H4,H3_ 3.2, *J*_H4,H5_ 8.2), 4.21 (1H, dd, H3, *J*_3,4_ 3.2, *J*_3,2_ 7.5), 4.24 (1H, q, H5, *J*_5,6_ = *J*_5,6′_ 4.0, *J*_5.4_ 8.2), 4.62 (1H, d, H2, *J*_2,3_ 7.5), δ_C_ (CDCl_3_, 100MHz) 26.0 (CH_3_), 26.5 (CH_3_), 26.7 (CH_3_), 27.3 (CH_3_), 52.6 (OCH_3_), 61.7 (C6), 75.5 (C2), 76.2 (C4), 77.3, 77.7 (C3 and C5), 109.8 (C(CH_3_)_2_), 111.7 (C(CH_3_)_2_), 171.0 (C1); *m*/*z* (ESI + ve): 343 ([M + Na]^+^, 100%).

### 3.6. d-Idose 6

#### 3.6.1. Method 1 (from **13**)

Silica gel-supported NaIO_4_ (4.50 g) was added portionwise to a vigorously stirred solution of the triol **13** (668 mg, 2.29 mmol) in dichloromethane (30 mL). After 2 h, TLC analysis (ethyl acetate) showed no remaining starting material (*R*_f_ 0.31) and formation of one elongated spot (*R*_f_ 0.56–0.65). The reaction mixture was filtered and the silica gel was thoroughly washed with dichloromethane (4 × 30 mL). The solvents were removed in vacuo to afford the crude aldehyde **14** (600 mg, 100%), which was dissolved in water (20 mL) and treated with DOWEX^®^ 50WX8-200 (~400 mg, prewashed with water). After stirring at rt for 24 h, TLC analysis (ethyl acetate) showed no remaining starting material and formation of a single product (baseline). The resin was filtered and washed with water. Removal of water in vacuo afforded D-idose **6** (400 mg, 97% from **13**; 53% from **11**) as a colorless syrup.

[α]_D_^20^ + 10.7 (*c* 0.55, water) (authentic sample: [α]_D_^20^ + 10.0 (*c* 0.80, water), [1] [α]_D_^17^ + 13.7 (*c* 2.47, water)], ^1^H, and ^13^C NMR are identical with those of authentic sample; HPLC showed purity 85%; After HPLC purification, purity reached to 100%; *m*/*z* (ESI + ve): 203 ([M + Na]^+^, 100%) HRMS *m*/*z* (ESI + ve): found 203.0524 ([M + Na]^+^), C_6_H_12_O_6_Na^+^ requires 203.0526.

#### 3.6.2. Method 2 (from **16**)

Diisobutylaluminum hydride (1.0 M in toluene, 4.05 mL, 4.05 mmol) was added dropwise to a solution of **16** (391 mg, 1.35 mmol) in dichloromethane (5 mL) at −78 °C. The reaction mixture was stirred at −78 °C for 2 h until TLC analysis (ethyl acetate) showed no remaining starting material (*R*_f_ 0.31) and the formation of one spot (*R*_f_ 0.56–0.65). Mass spectrometry also showed the formation of desired product peak ([M + MeOH + Na]^+^ 315) and disappearance of starting material peak ([M + Na]^+^ 313). The mixture was diluted with ethyl acetate (10 mL) and potassium sodium tartrate (sat., *aq*., 2 mL) was added. After stirring for 8 h, the reaction mixture was diluted with water (10 mL) and extracted with ethyl acetate (3 × 10 mL). The organic phase was dried (MgSO_4_) and filtered; then, the solvent was removed in vacuo to obtain an oil that was further purified by flash chromatography (cyclohexane/ethyl acetate 7:1 to 3:1) to yield crude aldehyde **14** as a syrup (267 mg, 76%). The crude aldehyde **14** was dissolved in water (20 mL) was treated with DOWEX^®^ 50WX8-200 (~300 mg, prewashed with water). After 24 h, TLC analysis (ethyl acetate) showed no remaining starting material and formation of a single product (baseline). The resin was filtered and washed with water. Removal of water in vacuo afforded d-idose **6** (185 mg, 76% from **16**; 21% from **11**) as a colorless syrup.

[α]_D_^20^ + 11.7 (*c* 0.65, water, eq), ^1^H and ^13^C NMR were identical to those produced by Method 1 above.

## 4. Conclusions

Neither d-idose nor any d-*ido*-furanosides/d-*ido*-pyranosides have been reported as natural products, so it may be that such idosides attached to steroids or alkaloids would not be subject to enzymic hydrolysis and provide a novel set of glycosylated—but enzymically stable—bioactive compounds. Repetition of the isolation of the pure cadmium salt of the *ido*-heptonate **2** by the method of Isbell and Frush [21] will surely be achieved by other workers and would allow the preparation in three efficient steps of the triol diacetonide **13** as a stable crystalline precursor for d-idose, merely by subsequent periodate cleavage and mild acid hydrolysis. Although the preparation from the *gluco*-heptononic acid **3** in this paper is rather longer, it provides practical multigram scalable access to a stable form of d-idose suitable either for investigation of the properties of the free sugar or the investigation of d-idosides of oligosaccharides and pharmacophores. Precursors for d-iduronic and d-idonic acids are also easily prepared. Even for such densely functionalized compounds with seven adjacent oxygen groups, acetonide is the only protection needed, as it is cheap, selective and often crystalline.

## Supplementary Materials

Supplementary material of ^1^H and ^13^C NMR spectra is provided online.
