# Supplementary material for: d-Idose, d-Iduronic Acid, and d-Idonic Acid from d-Glucose via Seven-Carbon Sugars
Source: Molecules. 2019 Oct 18;24(20):3758. doi: 10.3390/molecules24203758 (PMC6832733; doi:10.3390/molecules24203758)

SUPPLEMENTARY MATERIAL.

D-Idose, D-iduronic acid and D-idonic acid from D-glucose *via* seven carbon sugars

Zilei Liu,[a,c] Sarah F. Jenkinson,[a] Akihide Yoshihara,[b] Mark R. Wormald,[c] Ken Izumori,[b] George W. J. Fleet[a,b]*

[a] *Chemistry Research Laboratory, Department of Chemistry, University of Oxford, Oxford, OX1 3TA, UK*

[b] *International Institute of Rare Sugar Research and Education, Kagawa University, Miki, Kagawa 761-0795, Japan*

[c] *Oxford Glycobiology Institute, Department of Biochemistry, University of Oxford, Oxford, OX1 3QU, UK*

**george.fleet@chem.ox.ac.uk*

^{1}H and ^{13}C NMR Spectra of new compounds

Page 2: **Methyl 2,3:4,5:6,7-tri-*O*-isopropylidene-D-*glycero*-D-*ido*-heptonate 11**

Page 3: **Methyl 2,3:4,5-di-*O*-isopropylidene-D-*glycero*-D-*ido*-heptonate 12**

Page 4: **2,3:4,5-Di-*O*-isopropylidene-D-*glycero*-D-*ido*-heptitol 13**

Page 5: **Methyl 2,3:4,5-di-*O*-isopropylidene-D-*glycero*-D-idonate 16**

Page 6: **D-Idose 6**

Methyl 2,3:4,5:6,7-tri-*O*-isopropylidene-D-*glycero*-D-*ido*-heptonate 11

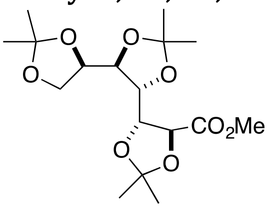

^{1}H NMR (CD_3OD, 400 MHz)

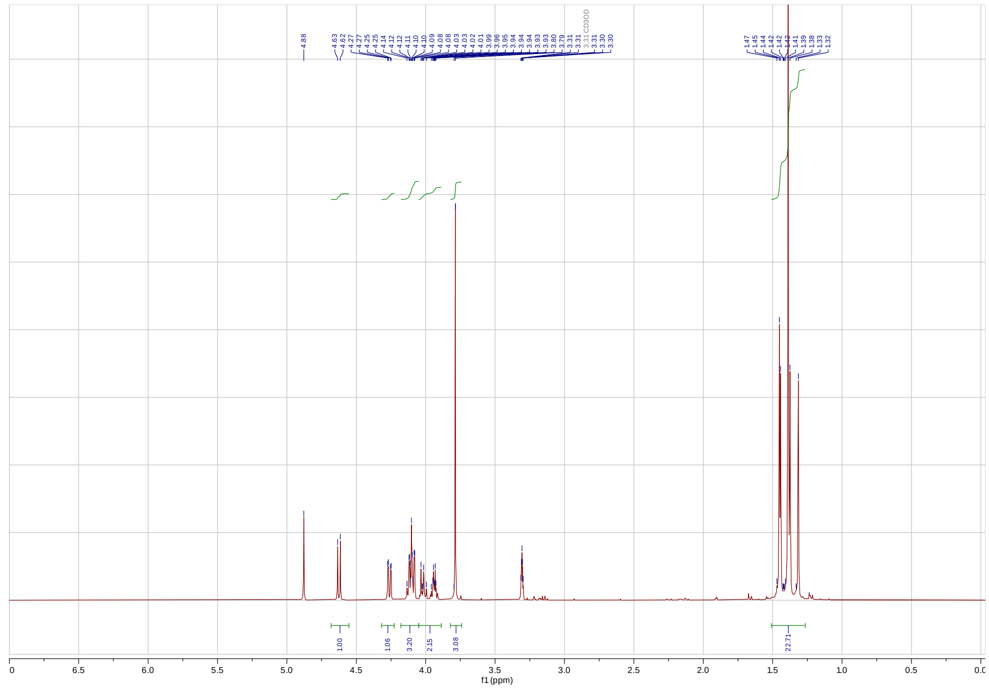

^{13}C NMR (CD_3OD, 100 MHz)

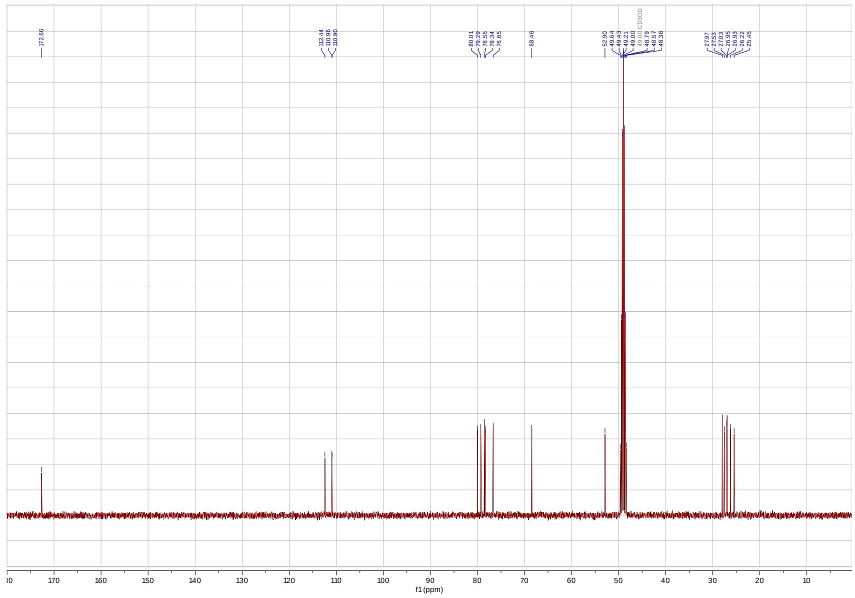

Methyl 2,3:4,5-di-*O*-isopropylidene-D-*glycero*-D-*ido*-heptonate 12

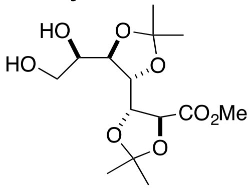

^{1}H NMR ($CDCl_3$, 400 MHz)

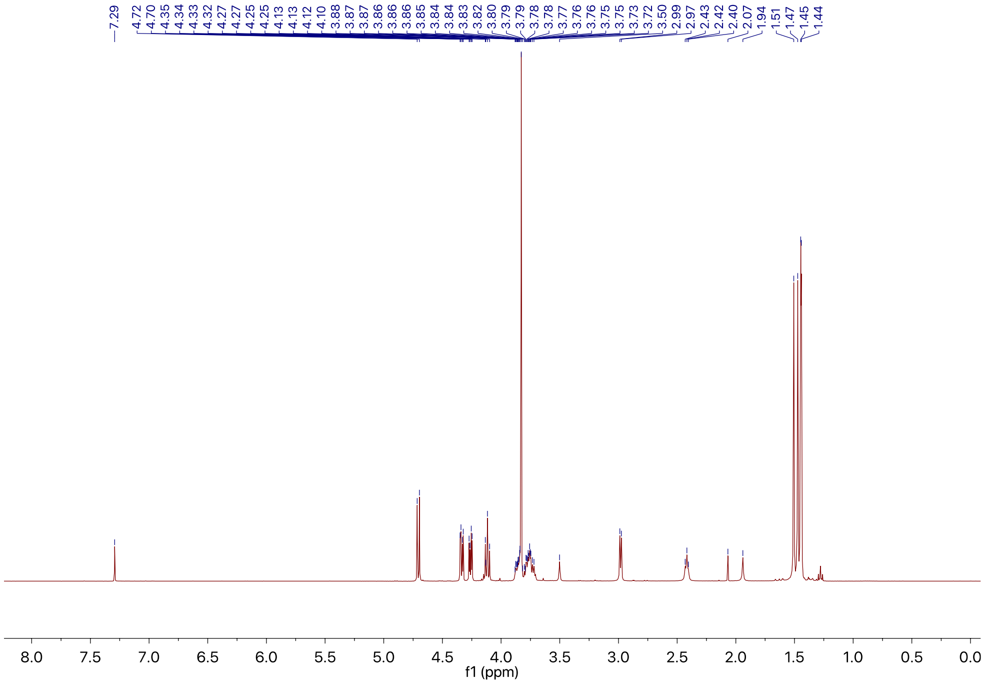

^{13}C NMR ($CDCl_3$, 100 MHz)

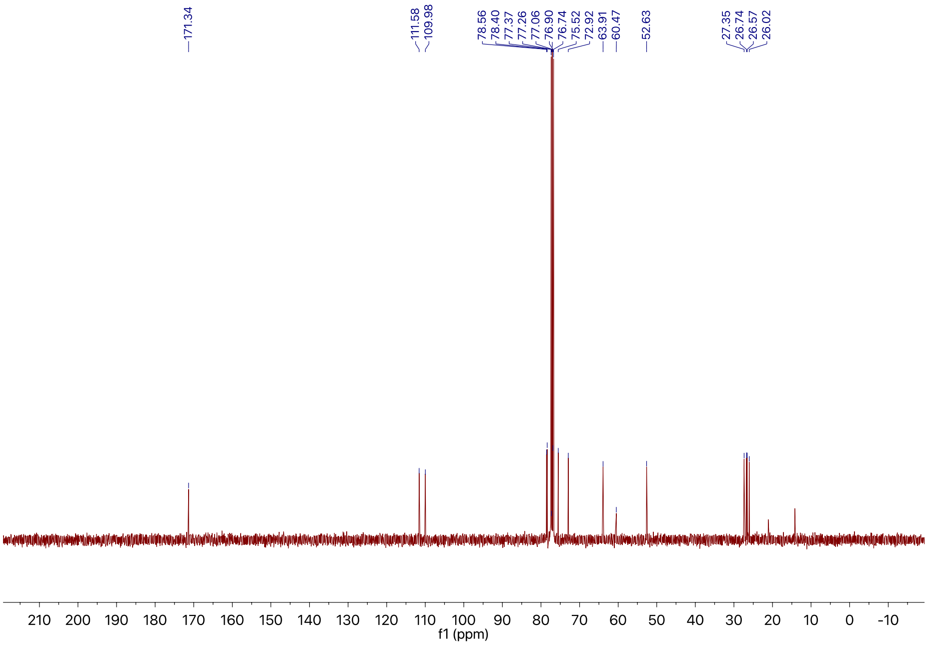

2,3:4,5-Di-*O*-isopropylidene-D-*glycero*-D-*ido*-heptitol 13

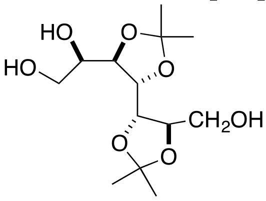

^{1}H NMR (CD_3OD, 400 MHz)

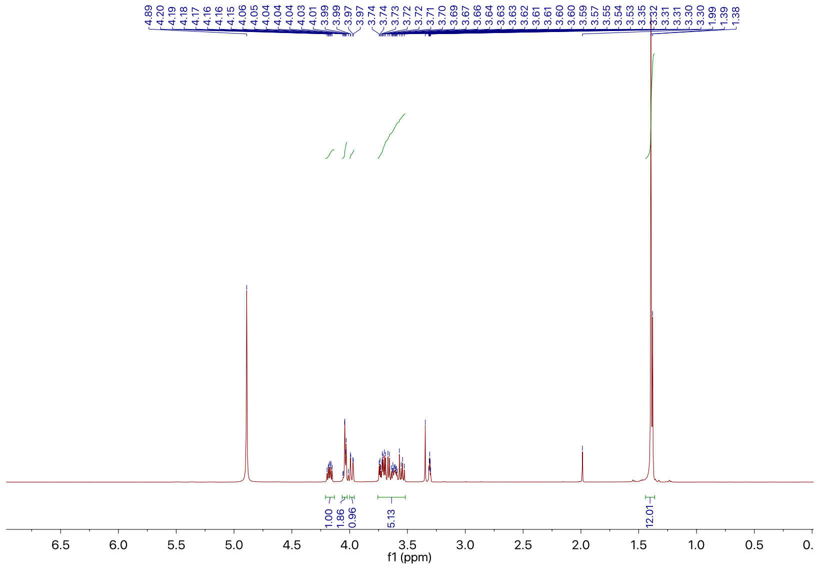

^{13}C NMR (CD_3OD, 100 MHz)

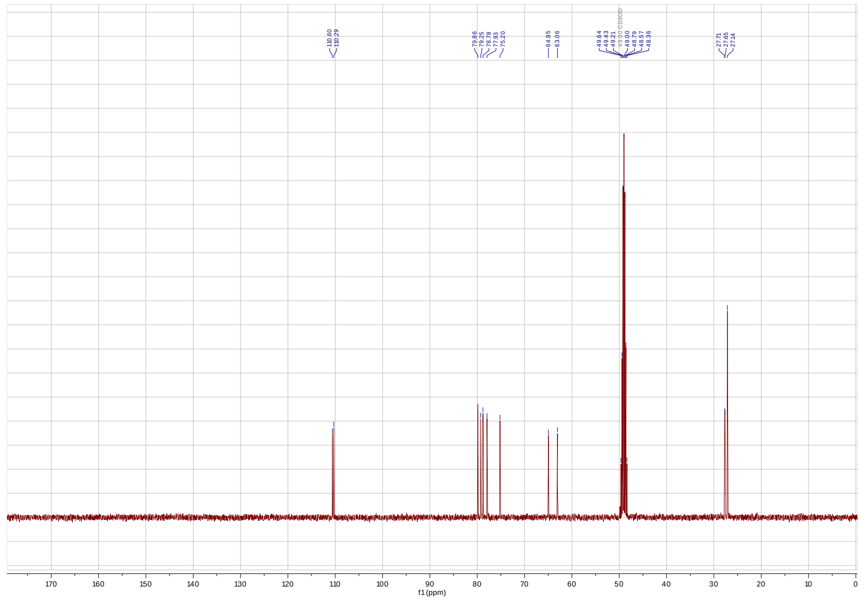

Methyl 2,3:4,5-di-*O*-isopropylidene-D-*glycero*-D-idonate 16

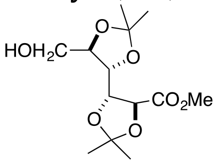

^{1}H NMR ($CDCl_3$, 400 MHz)

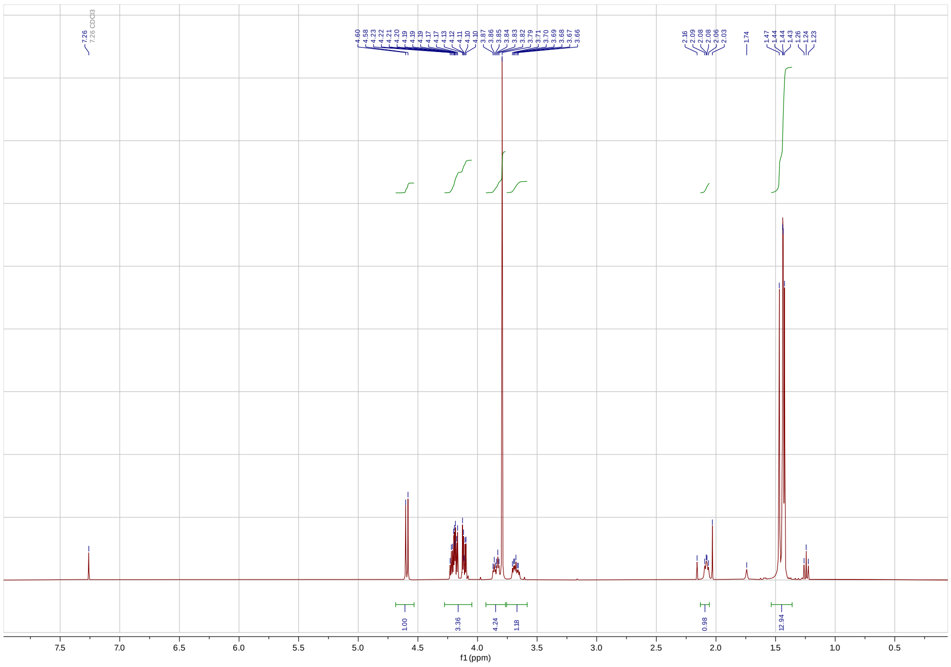

^{13}C NMR ($CDCl_3$, 100 MHz)

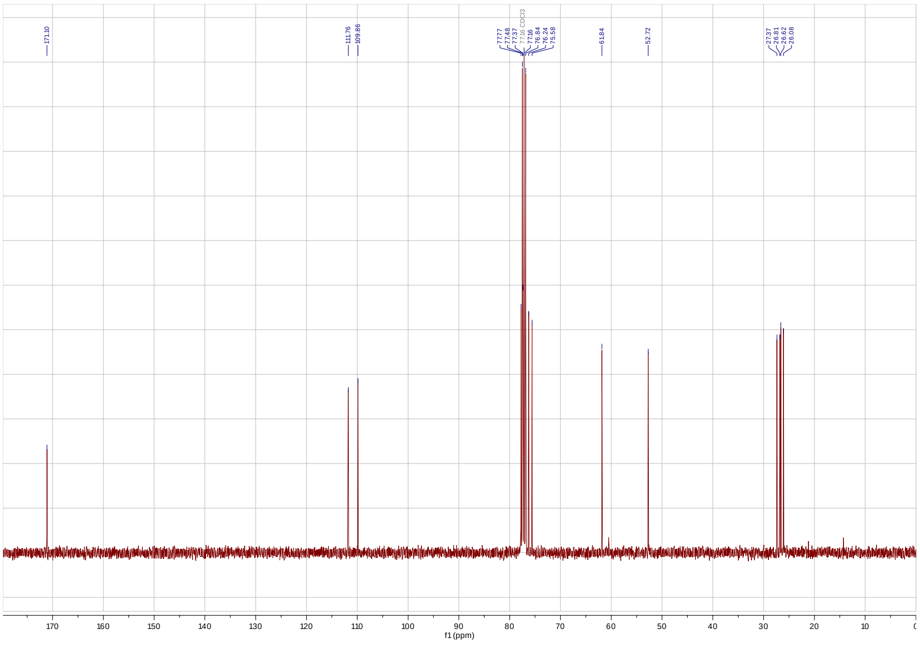

D-Idose 6

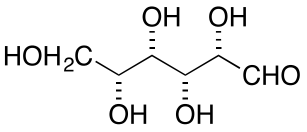

^1H NMR (D_2O, 400 MHz)

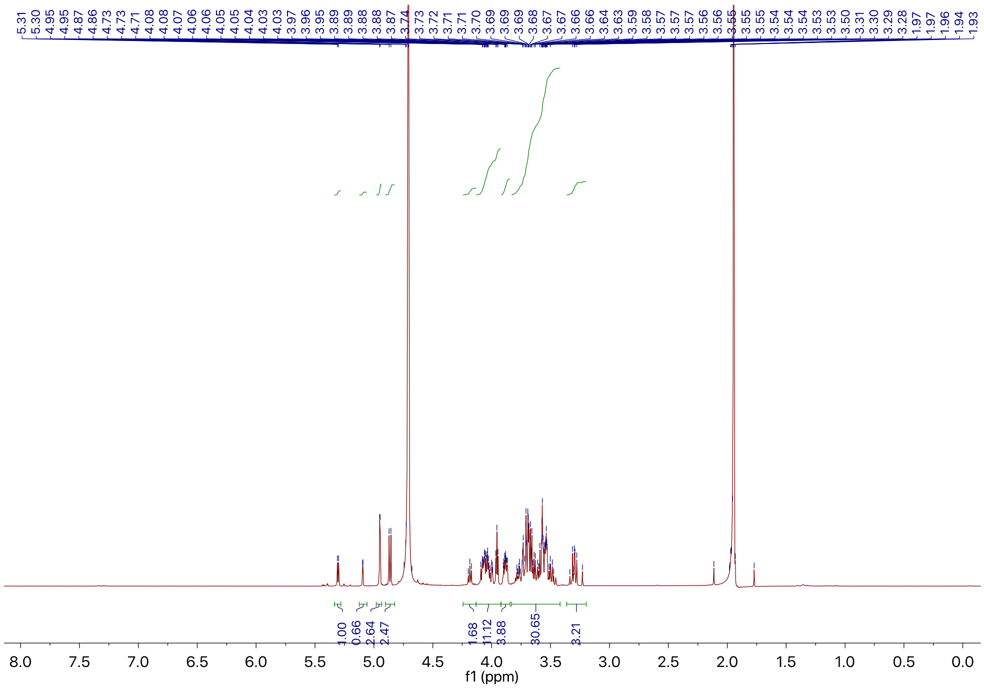

^{13}C NMR (D_2O, 100 MHz)

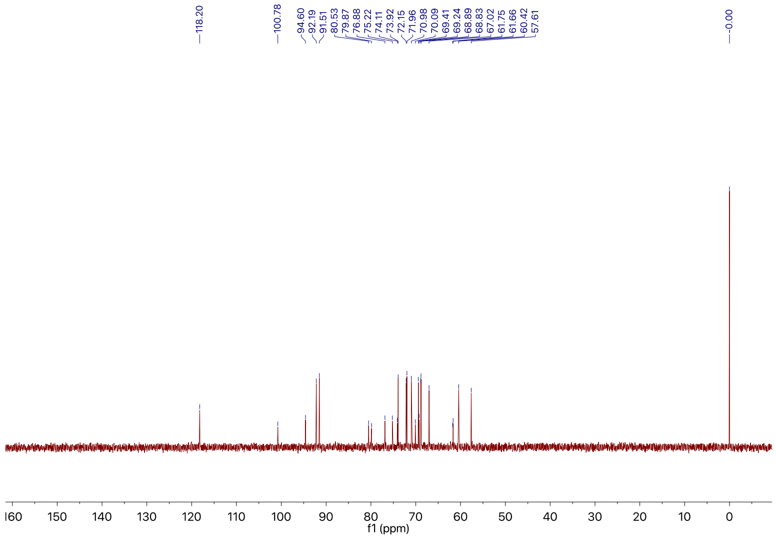

Supplement: Supplementary file 1 [file molecules-24-03758-s001.pdf]
